# Long-Term Follow-Up of Low-Risk Branch Duct IPMNs of the Pancreas: Watch for Main Pancreatic Duct Dilatation, and for How Long?

**DOI:** 10.1038/s41424-018-0065-9

**Published:** 2018-10-24

**Authors:** Thiruvengadam Muniraj, Harry R. Aslanian

**Affiliations:** Section of Digestive Disease, Yale School of Medicine, New Haven, CT USA

Pancreatic cancer continues to be recalcitrant with a 5-year relative survival rate of only 6%.^1^ It is usually discovered at an advanced stage and there currently are no proven screening or surveillance strategies.

Pancreatic cystic lesions are frequently incidentally diagnosed with prevalence rates of up to 15–40% in asymptomatic patients undergoing cross-sectional imaging.^2,3^ Branch-duct intraductal papillary mucinous neoplasms (BD-IPMN) are the most common incidentally noted type of cyst. The vast majority of BD-IPMNs do not progress to malignancy. Morphologic and/or biochemical predictive factors for malignant progression remain of great interest, to identify patients who would benefit from surveillance and/or surgical cyst resection. A minority of cysts are main-duct IPMN (MD-IPMN), which are typically readily distinguishable due to marked dilation of the main pancreas duct and referred for surgical resection owing to their high risk of malignancy.

Several guidelines have provided management recommendations, largely based on morphologic features. The International consensus (Fukuoka) guidelines by the International Association of Pancreatology (IAP)^4^, the evidence-based American Gastroenterological Association (AGA) guidelines^5^ and the European guidelines^6^ provide management guidance with emphasis on high-risk features, including cyst size greater than 3 cm, mural nodules, and dilation of the main pancreas duct. These guidelines have limitations including limited sensitivity and specificity for identifying high-risk cysts. All three guidelines differ in their recommendation on surveillance after 5 years for small asymptomatic cysts that do not have any high-risk stigmata (HRS) or worrisome features (WF). The IAP guidelines did not give any recommendation, the AGA guidelines based on a “very low-quality evidence” recommended discontinuation of surveillance after 5 years of “stability” and the European guidelines recommend annual follow-up for cysts > 15 mm. Studies by Kwong et al.^7^ and Crippa et al.^8^ have shown that there is a low rate of progression toward malignancy and development of new HRS/WFs in previously low risk cysts after 5, 10, or more years of stability. Balancing the costs of long-term surveillance imaging of typically low risk pancreas cysts and the morbidity and mortality of surgical resection versus the risk of malignant progression is very difficult. The AGA guidelines highlight the difficulty in proving that pancreas cyst surveillance accomplishes the ultimate goal of reducing pancreas cancer mortality.

Petrone et al.^12^ have attempted to elucidate the most predictive characteristics of malignancy in BD-IPMNs. They examined data from 167 patients with BD-IPMN referred to San Raffaele Scientific Institute, Milan, Italy between 2002 and 2016. Their retrospective analysis examined associations between the appearance of HRS or WF and the subsequent development of high-grade dysplasia or invasive carcinoma.

During the median 4.5-year follow-up, a high proportion of patients (58%) experienced a change in cyst characteristics. These were most frequently an increase in cyst size (35%) or the appearance of new lesions (33%). It is expected that there will be slow growth of BD-IPMN over time and when an increase in cyst size may indicate an increase of HGD or dysplasia remains uncertain. BD-IPMN are frequently multifocal and the recognition of new small cysts on imaging is typically of little concern and in practice may also be influenced the level of attention to detail or reporting of the interpreting radiologist and/or the quality of the imaging.

Worrisome features occurred in 26.3% of patients after median follow-up of 26 months. HRS developed in three patients (1.8%) after a median follow-up of 26 months and predicted malignancy in every case (100% specific). Nine patients underwent surgery, and malignancy was identified in seven (4.2% of 167 patients). Among the worrisome features, the authors found neither cyst size nor the rate of growth >5 mm/year as a significant predictor of malignancy. The presence of mural nodules and MPD dilation >5 mm, however, were both significantly associated with malignancy, the latter with an odds ratio of 24.5. Several malignancies occurred many years after initial diagnosis.

While this study is compelling in its long-term follow-up of a fairly large number of low-risk BD-IPMN, one can contemplate what the study of Petrone et al., adds to the large body of retrospective data on pancreas cysts. This study adds additional important data quantifying the risk of malignancy associated with the development of mural nodules and MPD dilation.

Petrone et al.^12^ confirms that the development of malignancy in low-risk branch duct IPMN cases is uncommon, however, it certainly occurs. Indeed, their data suggest that there is no time span after which a physician can be entirely confident that malignancy will not occur, despite the presence of a guideline recommendation. Pergolini et al.^11^ reported similar findings with 5.5% of patients developing malignancies 5 or more years after diagnosis of BD-IPMN, including 4.3% of patients who did not have any HRS/WF after 5 years.

The development of worrisome features in more than a quarter of patients with a median follow-up of ~2 years is remarkable. Petrone et al. showed that further projection of Kaplan–Meier curves suggests the development of additional worrisome features and malignancy, if these patients are followed for even longer time periods. Their results suggest that follow-up of IPMN cases should exceed 5 years, particularly in healthy patients without significant comorbidities.

It appears helpful to remind ourselves that the goal of pancreas cyst surveillance is to prolong life and reduce death from pancreas cancer. There is growing recognition that the morbidity and mortality of surgical resection and the likelihood of death from causes other than pancreas cancer should be factored in to decisions regarding long-term cyst surveillance.^9,10^ We agree with discontinuation of surveillance for patients who have multiple other comorbidities and are likely to die from nonpancreatic causes. While new generations of cyst fluid biomarkers may assist in risk stratification, at present, physicians treating patients with BD-IPMN need to keep a vigilant eye on their patients, well into the future, especially if they are young and healthy.
